# Metoprolol alleviates arginine vasopressin-induced cardiomyocyte hypertrophy by upregulating the AKT1–SERCA2 cascade in H9C2 cells

**DOI:** 10.1186/s13578-020-00434-y

**Published:** 2020-05-24

**Authors:** Jieqiong Zhao, Yonghong Lei, Yanping Yang, Haibo Gao, Zhongchao Gai, Xue Li

**Affiliations:** 1 Department of Cardiology, Tangdu Hospital, Air Force Medical University, Xi’an, 710038 Shaanxi People’s Republic of China; 2 Department of Plastic Surgery, General Hospital of Chinese PLA, Beijing, 100853 People’s Republic of China; 3 School of Food and Biological Engineering, Shaanxi University of Science and Technology, Xi’an, 710021 Shaanxi People’s Republic of China

**Keywords:** Arginine vasopressin, Metoprolol, AKT1, SERCA2, Cardiomyocyte hypertrophy

## Abstract

**Background:**

Arginine vasopressin (AVP) is elevated in patients with heart failure, and the increase in the AVP concentration in plasma is positively correlated with disease severity and mortality. Metoprolol (Met) is a beta blocker that is widely used in the clinic to treat pathological cardiac hypertrophy and to improve heart function. However, the specific mechanism by which Met alleviates AVP-induced pathological cardiac hypertrophy is still unknown. Our current study aimed to evaluate the inhibitory effects of Met on AVP-induced cardiomyocyte hypertrophy and the underlying mechanisms.

**Methods:**

AVP alone or AVP plus Met was added to the wild type or AKT1-overexpressing rat cardiac H9C2 cell line. The cell surface areas and ANP/BNP/β-MHC expressions were used to evaluate the levels of hypertrophy. Western bolting was used to analyze AKT1/P-AKT1, AKT2/P-AKT2, total AKT, SERCA2, and Phospholamban (PLN) expression. Fluo3-AM was used to measure the intracellular Ca^2+^ stores.

**Results:**

In the current study, we found that AKT1 but not AKT2 mediated the pathogenesis of AVP-induced cardiomyocyte hypertrophy. Sustained stimulation (48 h) with AVP led to hypertrophy in the H9C2 rat cardiomyocytes, resulting in the downregulation of AKT1 (0.48 fold compared to control) and SERCA2 (0.62 fold), the upregulation of PLN (1.32 fold), and the increase in the cytoplasmic calcium concentration (1.52 fold). In addition, AKT1 overexpression increased the expression of SERCA2 (1.34 fold) and decreased the expression of PLN (0.48 fold) in the H9C2 cells. Moreover, we found that Met could attenuate the AVP-induced changes in AKT1, SERCA2 and PLN expression and decreased the cytoplasmic calcium concentration in the H9C2 cells.

**Conclusions:**

Our results demonstrated that the AKT1–SERCA2 cascade served as an important regulatory pathway in AVP-induced pathological cardiac hypertrophy.

## Background

Pathological cardiac hypertrophy is associated with metabolic derangement, sarcomere disorganization, altered calcium handling, and sustained pathological hypertrophy led to arrhythmia, congestive heart failure, and sudden death [1, 2]. Many neurohumoral factors, such as catecholamines, angiotensin II and endothelin-1, can cause pathological cardiac hypertrophy [3, 4]. Arginine vasopressin (AVP) is released from the hypothalamus in response to changes in arterial pressure and plasma osmolality, and many previous studies have shown that AVP can induce peripheral vasoconstriction and cardiac hypertrophy [5–7].

The serine/threonine AKT protein kinases, also known as PKB or Rac, consists of three subtypes: AKT1, AKT2 and AKT3. AKT1 is extensively distributed in many tissues, AKT2 is mainly expressed in muscle and fat cells, and AKT3 is specifically expressed in testes and brain [8]. AKT regulates several biological processes related to cell growth and survival, differentiation, proliferation, growth and metabolism [9]. AKT is also an important contributor to cardiovascular disease due to its role in cardiac growth, angiogenesis, and cardiac hypertrophy. Hormones and cytokines are the classic activators of AKT signaling [10], neurohormonal factors accompanying with continuously overloaded biomechanical stress induce multiple signaling pathways such as PKC, MAPK and AKT pathway in pathological hypertrophy [2].

Pathological cardiac hypertrophy is directly related to myocardial contractility [11]. Increased intracellular free calcium is the main cause of myocardial cell contraction [12]. In hypertrophic cardiomyocytes, intracellular systolic Ca^2+^ is decreased, and diastolic Ca^2+^ is increased, which subsequently triggers a series of pathophysiological reactions. The reserve of Ca^2+^ in the sarcoplasmic reticulum (SR) is the basic factor that affects myocardial contraction. Studies have shown that heart failure (HF), myocardial ischemia and other cardiac diseases are accompanied by decreased Ca^2+^ storage in the SR [13–15]. Sarcoplasmic/endoplasmic reticulum Ca^2+^-ATPase (SERCA) is a type of cardiac SR Ca^2+^-ATP enzyme that plays an important role in the regulation of calcium homeostasis in cardiac myocytes [13, 16]. During diastole, the intracellular calcium in cardiomyocytes is mostly reabsorbed into the SR through SERCA2 in the sarcoplasmic reticulum membrane, and phospholamban (PLN) plays a major role in regulating SERCA2 activity. When PLN is dephosphorylated, PLN binds to SERCA2 to form the PLN–SERCA2 complex, which reduces the affinity of SERCA2 for Ca^2+^. When PLN is phosphorylated, the PLN–SERCA2 complex dissociates, and SERCA2 regains its Ca^2+^-binding ability [17].

Metoprolol (Met) is a β-blocker widely used to inhibit cardiac hypertrophy and to improve cardiac function [18, 19]. Nebivolol, which is a third-generation β-blocker, has antihypertrophic effects in models of neonatal cardiomyocyte hypertrophy [20]. AKT is involved in the diabetic and antiapoptotic effects of β-adrenergic stimulation [21, 22]. However, whether AKT mediates the inhibitory effects of Met during cardiac hypertrophy remains unknown. In addition, the specific mechanism by which Met alleviates cardiomyocyte hypertrophy urgently needs to be investigated. Therefore, we carried out this study to identify the potential players that mediate the AVP-induced hypertrophy and the protective effect of Met. Our results revealed the relationship between Met and the AKT1–SERCA2 signaling pathway in AVP-induced pathological cardiac hypertrophy.

## Results

### Hypertrophic effect of AVP on H9C2 cells

**Fig. 1:**
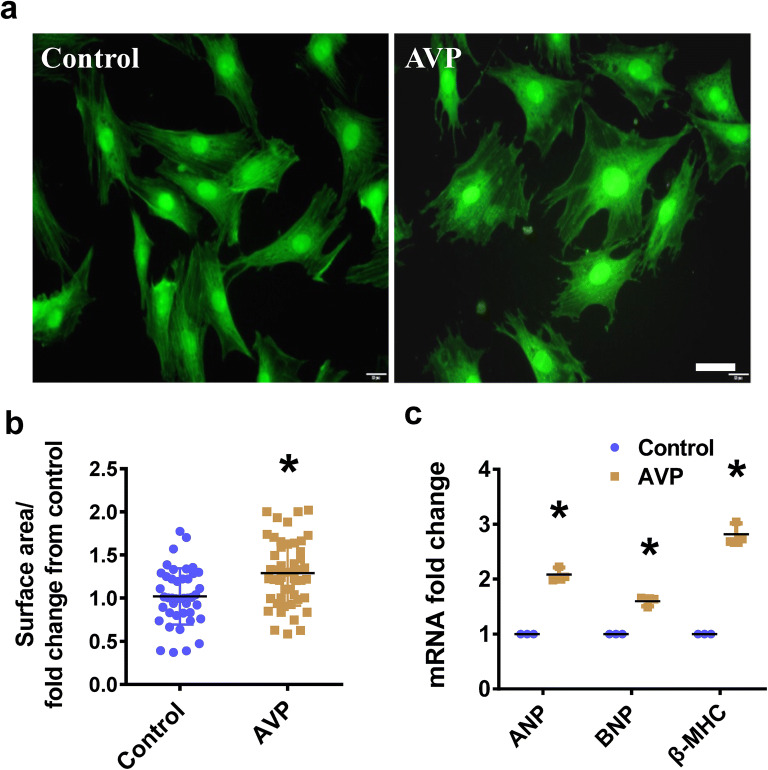
AVP caused a hypertrophic phenotype in the H9C2 cells. **a** F-actin staining (scale bar = 200 μm) was performed to determine the hypertrophic growth of the H9C2 cells treated with AVP. **b** Quantification of the surface areas of the AVP-stimulated H9C2 cells. **c** The mRNA levels of ANP and BNP and β-MHC were measured in the H9C2 cells stimulated with AVP. All the data are presented as the mean ± S.E.M. of at least three independent experiments. *P < 0.05 compared with the Control (Con)

We initially established a model of cardiomyocyte hypertrophy by exposing H9C2 cells to AVP for 48 h. The rhodamine-phalloidin (F-actin) staining and quantification results showed that the cell surface area increased (average of 1.265-fold compared to control-treated cells) when the cells were treated with AVP (Fig. 1a, b). The mRNA expression levels of the cardiomyocyte hypertrophy markers atrial natriuretic peptide (ANP), B-type natriuretic peptide (BNP) and beta-myosin heavy chain (β-MHC) were greatly increased in the AVP-stimulated H9C2 cells (Fig. 1c).

### AKT1 potentially participated in AVP-induced hypertrophy in H9C2 cells

**Fig. 2:**
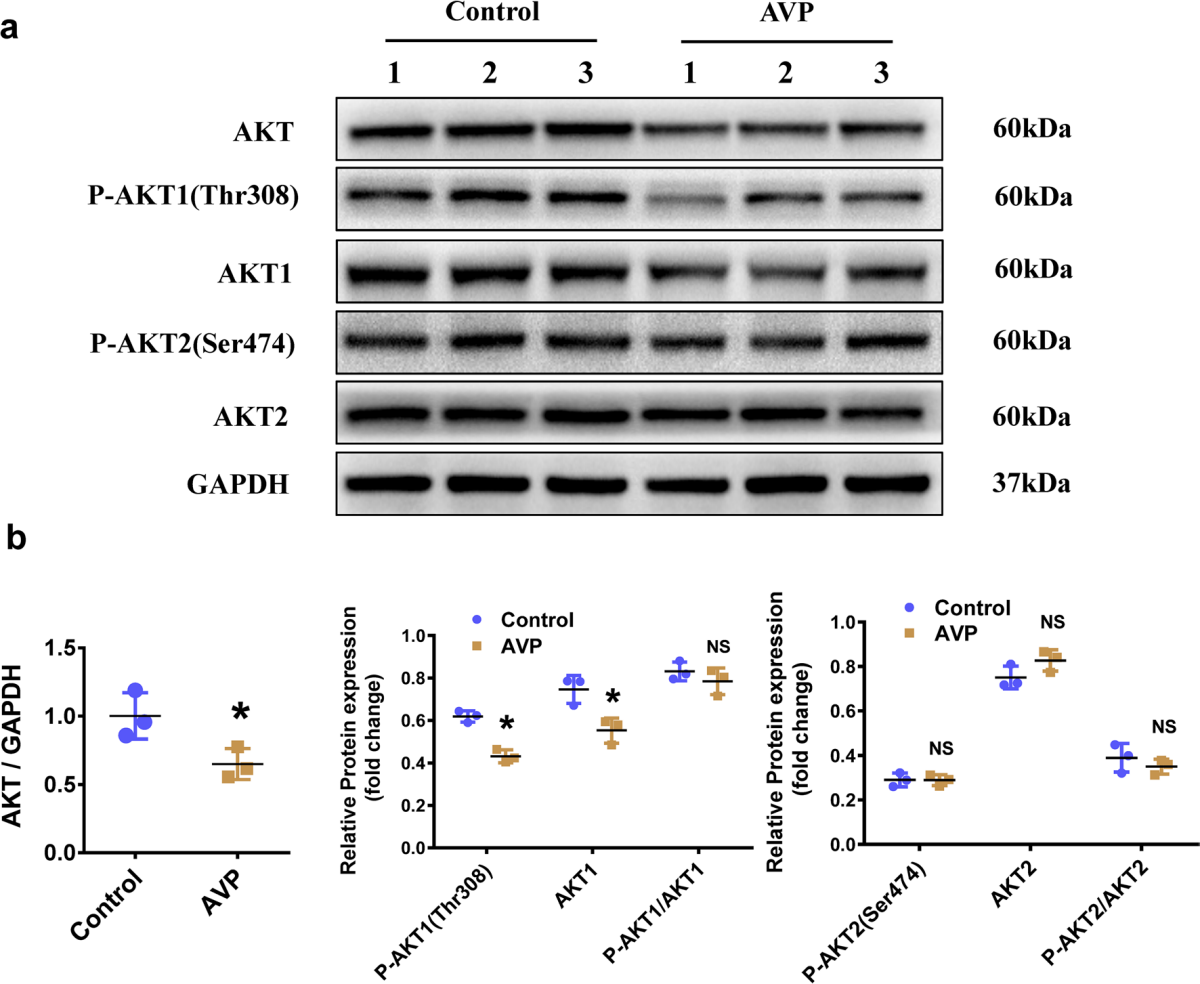
AKT1 was downregulated in the AVP-induced hypertrophic H9C2 cells. **a** The expression of total AKT, AKT1, phosphorylated AKT1 (P-AKT1), AKT2, and P-AKT2 in the H9C2 cells treated with AVP was determined by western blot analyses. GAPDH was taken as the loading control. **b** Quantification of the protein expression of total AKT, AKT1, P-AKT1, AKT2, and P-AKT2 in the AVP-induced H9C2 cardiomyocytes. All data are presented as the mean ± S.E.M. of three independent experiments (labeled as 1, 2 and 3). * Indicates P < 0.05, *NS* not significant compared with the Control

To explore the underlying mechanisms involved in AVP-induced cardiomyocyte hypertrophy in H9C2 cells, we used western blot to analyze the expression levels of relevant proteins. Compared with the control treatments, exposure to AVP for 48 h led to significantly decreased levels of total AKT (0.65-fold), P-AKT1-Thr308 (0.70-fold) and AKT1 (0.74-fold), while the levels of AKT2 and P-AKT2(Ser474) were not altered (Fig. 2a, b, Additional file 1: Fig. S1).

### AKT1 overexpression attenuated AVP-induced cardiomyocyte hypertrophy

**Fig. 3:**
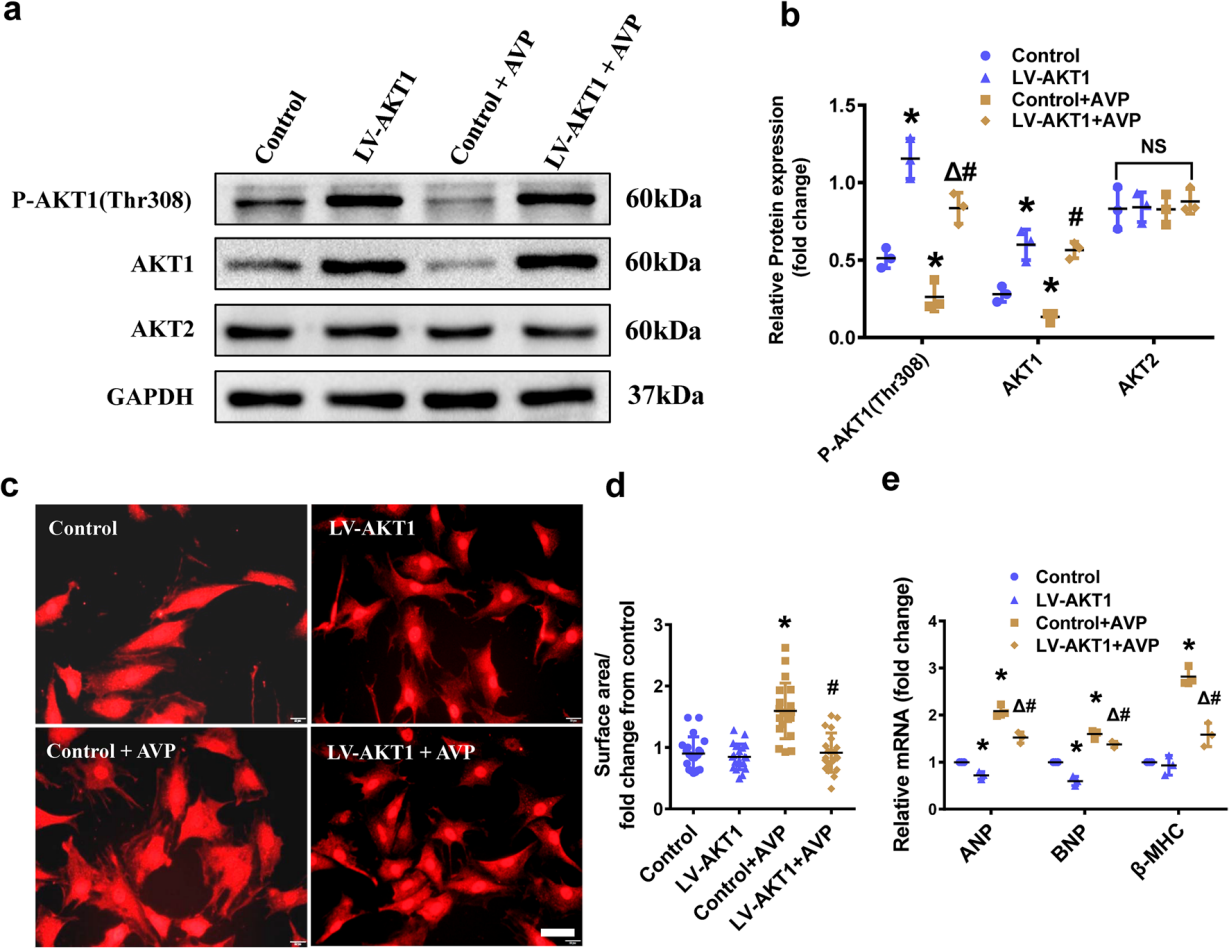
AKT1 overexpression inhibited the AVP-induced H9C2 hypertrophy. **a**, **b** The protein expression and quantification of P-AKT1(Thr308), AKT1 and AKT2 levels in the AKT1 overexpressing H9C2 stable strain, GAPDH was taken as the internal control. **c**, **d** α-Actinin staining (scale bar = 20 μm) was performed to determine the hypertrophic levels of the Control and AKT1 overexpressing H9C2 cells treated with AVP. **e** The mRNA levels of ANP, BNP and β-MHC were measured in the AKT1 overexpressing H9C2 stable strain. All the data are presented as the mean ± S.E.M. of at least three independent experiments. *^,#,Δ^P < 0.05; *NS* not significant. *Compared with Con; ^#^compared with Con + AVP; ^Δ^compared with LV-AKT1

To further investigate the effect of AKT1 on myocardial hypertrophy induced by AVP, we constructed an AKT1 overexpressing stable H9C2 strain (Lentivirus-AKT1). Western blot analysis showed that P-AKT1-Thr308 and AKT1 were markedly expressing in the AKT1 overexpressing stable strain with or without AVP stimulation, while the AKT2 was not altered (Fig. 3a, b). There was a 1.92-fold downregulation of the surface areas of AVP-treated H9C2 cardiomyocytes overexpressing AKT1 (Fig. 3c, d). The mRNA levels of ANP, BNP and β-MHC were also markedly decreased when AKT1 was overexpressed (Fig. 3e).

### AKT1 mediated AVP-induced cardiomyocyte hypertrophy through SERCA2/PLN

**Fig. 4:**
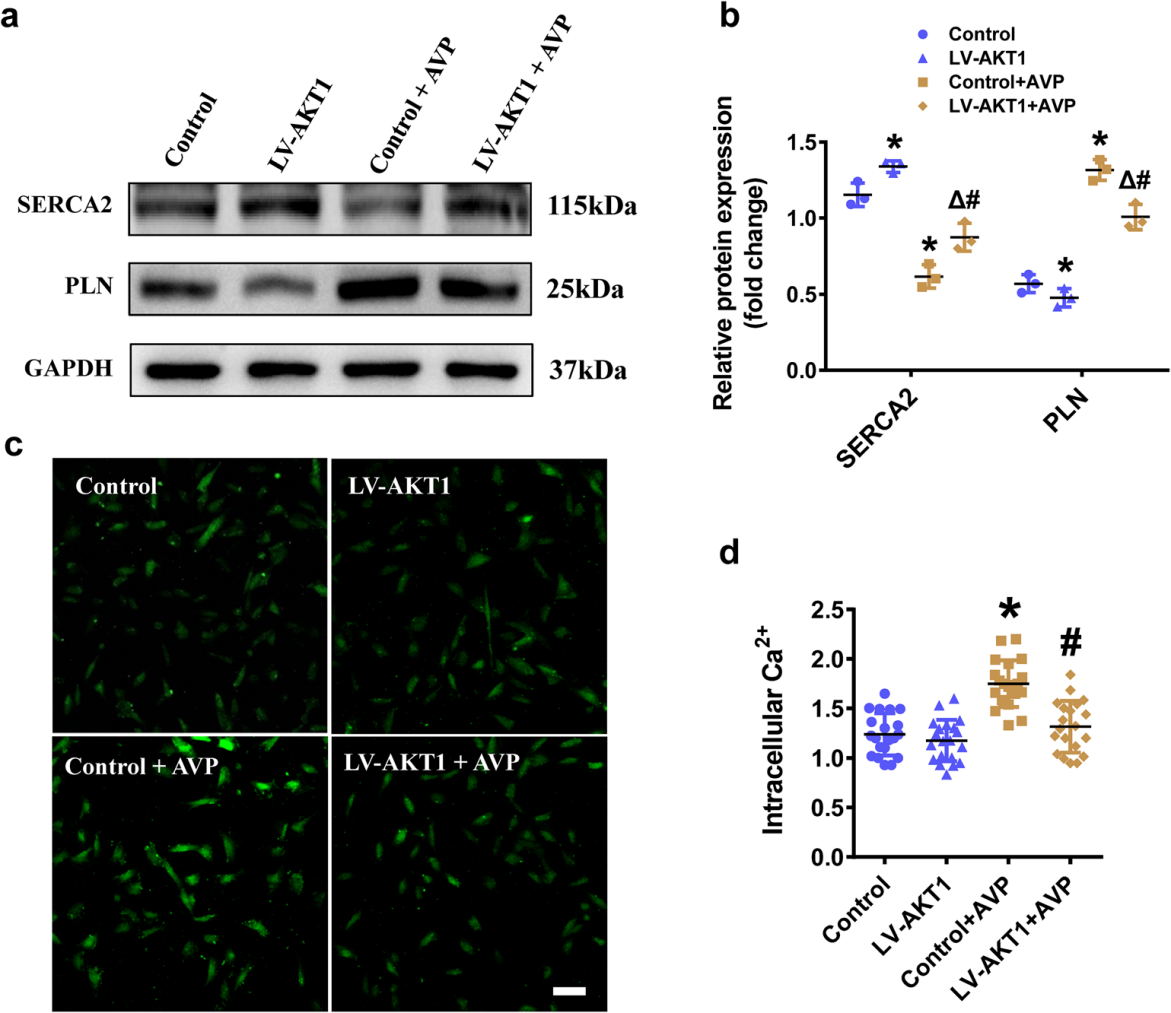
AKT1 overexpression upregulated the protein expression of SERCA2 and downregulated the protein expression of PLN. **a**, **b** The protein expression and quantification of the SERCA2 and PLN in the AKT1 overexpressing stable strain. GAPDH was used as the loading control. **c**, **d** Fluo-3/AM was used to measure the intracellular calcium concentration. The fluorescence (scale bar = 100 μm) and quantification of the calcium concentration in the cells. All the data are presented as the mean ± S.E.M. of at least three independent experiments. *^, #,Δ^Both indicates P < 0.05. *Compared with Control; ^#^compared with Control + AVP; ^Δ^compared with LV-AKT1

Studies were performed to gain further insight into the mechanism of cardiomyocyte hypertrophy when AKT1 was overexpressed. The protein expression of SERCA2 was significantly decreased, and the expression of PLN was increased in cardiomyocytes chronically treated with AVP compared with untreated cardiomyocytes. Moreover, SERCA2 expression was upregulated and PLN was downregulated when AKT1 was overexpressed. In AKT1 overexpressing H9C2 cells, the effects of AVP on SERCA2 and PLN expression were significantly attenuated (Fig. 4a, b, Additional file 1: Fig. S2). Additionally, we examined the intracellular calcium stores, and found that the intracellular Ca^2+^ concentration was significantly increased in response to AVP treatment, while this effect on the intracellular Ca^2+^ concentration was almost eliminated in AVP-treated H9C2 cells overexpressing AKT1(Fig. 4c, d).

### Metoprolol alleviated AVP-induced cardiomyocyte hypertrophy by upregulating the AKT1–SERCA2 cascade

**Fig. 5:**
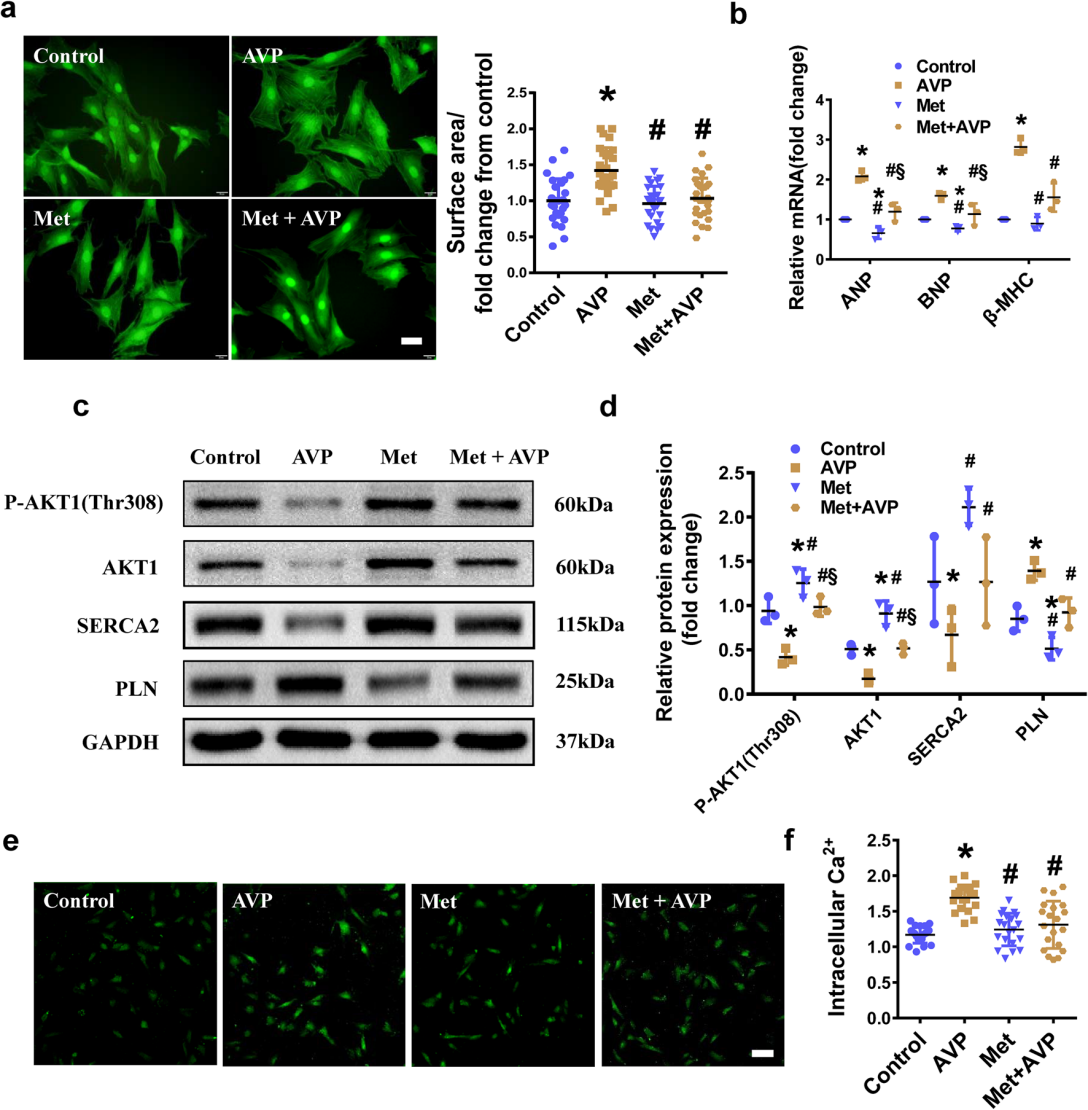
Metoprolol improved the AVP-induced decrease in the protein expression levels of AKT1 and SERCA2. **a** Left panel, F-actin staining (scale bar = 200 μm) was performed to measure the hypertrophic growth of the H9C2 cells treated with AVP, Met, and Met + AVP. Right panel, Quantification of the surface area of the H9C2 cells induced with AVP, Met, and Met + AVP. **b** The mRNA levels of ANP, BNP and β-MHC were measured in H9C2 cells treated with AVP, Met, and Met + AVP. **c**, **d** The protein expression and quantification of the P-AKT1(Thr308), AKT1, SERCA2 and PLN levels in the AVP-, Met-, and Met + AVP-treated H9C2 cells, GAPDH was selected as the loading control. **e**, **f** Fluo-3/AM was used to measure intracellular calcium concentration. The fluorescence (scale bar = 100 μm) and quantification of the calcium concentration in the AVP-, Met-, and Met + AVP-induced H9C2 cells. All the data are presented as the mean ± S.E.M. of at least three independent experiments. ^*,#,§^Both indicates P < 0.05; *compared with Control; ^#^compared with AVP; ^§^compared with Met

To examine the protective effects of Met on H9C2 cardiomyocytes exposed to AVP, and to investigate the mechanisms underlying this protective effect of Met, we performed a series of chemical treatment assays. We found that Met inhibited the AVP-induced increases in the H9C2 cell surface area (Fig. 5a) and the ANP, BNP and β-MHC mRNA levels (Fig. 5b), improved the AVP-induced decreases in the P-AKT1(Thr308), AKT1 and SERCA2 protein levels, and inhibited the AVP-induced increase in PLN protein level (Fig. 5c, d). The intracellular calcium concentration in the Met-treated group was decreased compared to that in the AVP-treated group (Fig. 5e, f).

## Discussion

Cardiac enlargement caused by hypertension stress, myocardial injury or neurohumoral hyperactivity is related to cardiac dysfunction, and this condition is known as pathological hypertrophy [2]. AVP is known to induce cellular pathological hypertrophy, as determined by increased cell surface area and elevated of ANP, BNP and β-MHC expression, and our results are consistent with previous studies [7, 23]. The known mechanisms of pathological cardiac hypertrophy include calcineurin-nuclear factor of activated T cell (NFAT) signaling, β-adrenergic receptor signaling, Ca^2+^/calmodulin-dependent kinase II signaling, cGMP/protein kinase G signaling, protein kinase C and mitogen-activated protein kinase signaling, and insulin receptor (IR)/AKT signaling [2]. AKT is an essential protein kinase that exerts important functions in numerous biological processes. The protein levels of P-AKT(Ser473), P-S6K1 and 4E-BP1 in hypertrophic hearts were reduced 8 weeks after TAC, indicating that pathological hypertrophy downregulates the AKT/mTOR pathway [24]. AKT1 and AKT2 are the major subtypes of AKT kinase expressed in the heart [25]. Our results revealed that the levels of total and phosphorylation AKT1 were both downregulated in AVP-induced pathological hypertrophic cells but that the levels of AKT2 were not altered. Furthermore, AKT1 overexpression inhibited the AVP-induced increases in the H9C2 cell surface area and the ANP, BNP and β-MHC mRNA expression, thus alleviating AVP-induced cardiomyocyte hypertrophy. These results indicate that the specific subtype that plays an important role in mediating pathological hypertrophy is AKT1, not AKT2.

Many studies have shown that the function of calcium pump was impaired due to the reduction in the SERCA2 protein levels in hypertrophic cells, and that the uptake of Ca^2+^ from the cytoplasm to the SR was reduced, lead to cytoplasmic calcium overload, and further resulting in diastolic calcium transient extension and decreased myocardial contractility [26–28]. Due to the decrease in the Ca^2+^ levels transported by SERCA2 into the SR, insufficient calcium is stored in the SR, resulting in reduced calcium release during the systolic period and myocardial contractile dysfunction [29–31]. However, the mechanism by which AVP affects Ca^2+^ has not been well studied. We found that the expression of SERCA2 was decreased in hypertrophic cardiomyocytes, and this result was consistent with the hypertrophic phenotype. PLN is an inhibitor of Ca-ATPases and can inhibit Ca^2+^ transport by interacting with Ca-ATPase, such as SERCA2 [32]. In this study, PLN expression was significantly upregulated in AVP-induced hypertrophy, this increase in PLN led to greater SERCA2 inhibition, and then, the transport of intracellular Ca^2+^ was blocked. These results suggested that AVP inhibited cardiac function by regulating the expression levels of SERCA2 and PLN. Furthermore, we showed that the expression level of AKT1 positively correlated with the expression level of SERCA2 and the concentration of intracellular Ca^2+^. The upregulation of AKT1 significantly attenuated the changes in the expression of SERCA2 and PLN caused by AVP treatment in H9C2 cells. All these findings indicate that AKT1–SERCA2-Ca^2+^ signaling is an underlying pathway involved in AVP-mediated inhibition of heart function. AKT kinase has been used as a potential gene therapeutic target for various diseases, such as hyperinsulinemia, diabetes, and hepatocellular carcinoma [33–35]. Our results also suggest that AKT1 can be taken as a possible gene therapeutic target for the treatment of pathological cardiac hypertrophy and to improve heart function. Although our study suggests that the AKT1–SERCA2 cascade exerts important functions in AVP-induced pathological cardiac hypertrophy, there may be some differences with those in vivo. H9C2 cells were originally derived from embryonic cardiac tissue, but cardiac myocytes in vivo have a well-defined structure and are subjected to mechanical overload in heart failure. To better understand the mechanisms involved in cardiac hypertrophy, additional studies are needed in the future to evaluate these findings and to identify more specific mechanisms in vivo.

Metoprolol is a selective β1-adrenoceptor inhibitor, that has been widely used to improve heart function [36, 37]. Many studies have shown that long-term treatment with Met could downregulate collagen I and III mRNA levels, and modulate stem cell characteristics to prevent progressive pathological remodeling, in addition, Met could improve the Ca^2+^ handling and contractility of right ventricular (RV) myocytes isolated from monocrotaline (MCT)-treated rats [18, 38–40]. In embryonic development, β-adrenoceptor stimulation leads to decreased AKT phosphorylation [41]. During ischemia–reperfusion, the activation of PI3K–Akt signaling contributes to the cardioprotective effects of Ca^2+^ antagonists and β-blocker [42]. However, the relationship between AKT1 and Met in AVP-induced hypertrophy is still unclear. In the current study, we found that Met significantly alleviated AVP-induced hypertrophy, and further experiments revealed that the levels of AKT1 and phosphorylated AKT1 were both increased in AVP-induced cardiomyocyte hypertrophy in H9C2 cells. These results described above indicated that AKT1 mediated the cardiac protective function of Met. Furthermore, the expression of SERCA2 was upregulated after Met treatment of AVP-induced hypertrophic H9C2 cells, in contrast, PLN, which is an inhibitor of SERCA2, was greatly decreased after Met treatment.

**Fig. 6:**
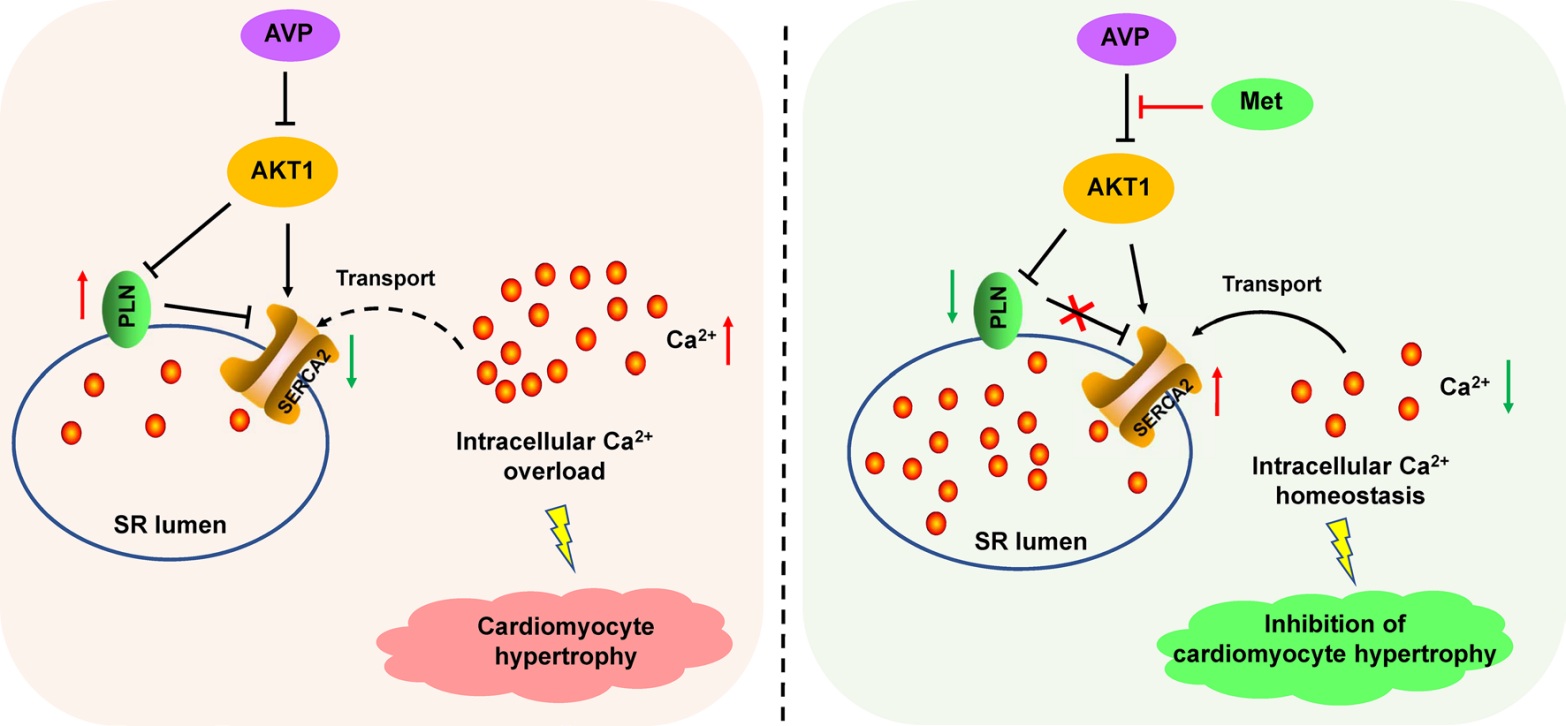
Working model of the mechanism by which Met alleviated AVP-induced cardiomyocyte hypertrophy through the AKT1–SERCA2 cascade. SERCA2 pumps Ca^2+^ into the sarcoplasmic reticulum and maintains the dynamic equilibrium of intracellular Ca^2+^. (left) In response to AVP induction, AKT1 is downregulated, and its inhibition of PLN and upregulation of SERCA2 are both attenuated. Moreover, the upregulation of PLN further inhibits Ca^2+^ transport through SERCA2. The intracellular Ca^2+^ concentration continues to rise and eventually leads to cardiomyocyte hypertrophy. (right) In H9C2 cells with AVP-induced hypertrophy, the inhibitory effect of AVP on AKT1 is attenuated by metoprolol. AKT1 inhibits PLN and upregulates SERCA2, and the excessive Ca^2+^ in the cytosol is pumped into the sarcoplasmic reticulum. Ultimately, the cardiomyocyte hypertrophy is alleviated

In response to AVP induction, AKT1 was downregulated, and its inhibition of PLN and upregulation of SERCA2 are both attenuated. These changes of PLN and SERCA2 further lead to the overload of intracellular Ca^2+^. Many studies have suggested that intracellular Ca^2+^ dynamics are closely linked with physiological cardiac states and intracellular Ca^2+^ overload could lead to cardiac hypertrophy [43–45]. Under the Met treatment, the upregulation of SERCA2 and the downregulation of PLN enabled effective intracellular Ca^2+^ transport, promoted intracellular Ca^2+^ homeostasis, and eventually alleviated AVP-induced hypertrophy (Fig. 6). Taken together, our results showed that Met alleviated AVP-induced hypertrophy through the AKT1–SERCA2 signaling pathway.

## Conclusions

In the present study, we demonstrated that the AKT1–SERCA2 cascade served as an important regulatory pathway in AVP-induced cardiomyocyte hypertrophy. First, we found that AKT1, not AKT2, mediated the pathogenesis of AVP-induced cardiomyocyte hypertrophy. Second, AVP induced hypertrophy in H9C2 cells by regulating the expression of SERCA2 and the transport of intracellular Ca^2+^. Third, Met alleviated AVP-induced hypertrophy through the regulation of the AKT-SERCA2 cascade.

## Materials and methods

### Antibodies

The primary antibodies specific for GAPDH (Catalog number ab9485), P-AKT1(Thr308) (Catalog number ab66134), P-AKT2 (Catalog number ab38513), AKT2 (Catalog number ab66129) and SERCA2 (Catalog number ab3625) were purchased from Abcam (Cambridge, MA, USA); the primary antibody against AKT1 (Catalog number TA504230) was purchased from DBA Acris Antibodies (Rockville, MD, USA); and the primary antibody against AKT (Catalog number CS4685) and PLN (Catalog number #14562) were obtained from Cell Signaling Technology (Boston, MA, USA). The HRP-conjugated secondary antibodies (Catalog numbers ab6721 and ab6885) and a FITC-conjugated secondary antibody (Catalog number ab6717) were purchased from Abcam.

### Materials

[Arg8]-vasopressin acetate salt (AVP) (Catalog number V9879) and metoprolol (Met) (Catalog number M1830000) were purchased from Sigma Aldrich (St. Louis, MO, USA). High-glucose DMEM (Catalog number SH30243.01), phosphate-buffered saline (PBS) (Catalog number SH30256.01) and trypsin (Catalog number SH30042.01) were purchased from GE Healthcare (Logan, UT, USA), and fetal bovine serum (FBS) (Catalog number 10270106) and Fluo-3/AM (Catalog number F1241) were obtained from Thermo Fisher Scientific (Waltham, MA, USA). Culture dishes (Catalog numbers 430166 and 430167) were purchased from Corning Costar Company (Cambridge, MA, USA). Confocal Petri dishes (Catalog number 801002) were obtained from NEST (Wuxi, Jiangsu, China).

### Cell culture

The H9C2 cell line (Catalog number GNR 5) was obtained from the Cell Bank of the Chinese Academy of Sciences (Shanghai, China), and cells of passage numbers 10–15 were used for all the experiments. The cells were cultured in high-glucose DMEM supplemented with 10% FBS and 1% penicillin–streptomycin in a 37 °C incubator with 5% CO_2_. The cells were treated with the chemicals (10 μmol/L AVP or Met) under the same conditions as those for growth.

### RT-PCR

Real-time quantitative PCR analysis was performed as previously described [46]. The primer sequences are described in Table 1. Total RNA was isolated with the TRIzol (Catalog number 15596026, Life Technologies, California, USA) method, and then DNase was used to remove internal DNA contamination, as previously described. Random primers were used in the reverse transcription reactions to obtain the first-strand cDNA, and the SYBR Green qPCR Master Mix (Catalog number FP202, Tiangen, Beijing, China) was used to amplify the target fragments. GAPDH cDNA amplification was used as an internal control to analyze the results. The general reaction procedure included an initial denaturing step at 95 °C for 15 min followed by 39 cycles of denaturing at 95 °C for 10 s, annealing at 58.2 °C for 30 s, and extension at 72 °C for 20 s, with a plate read at 95 °C for 5 s. Three independent replicates were performed for each experimental group, and IBM SPSS (version 19) software was used to analyze the differences in expression.

**Table 1:** Primers used for real-time qPCR analyses in this study

Target genes	Primer sequences	
Rat		
GAPDH	Forward	5′-ACAGCAACAGGGTGGTGGAC-3′
	Reverse	5′-TTTGAGGGTGCAGCGAACTT-3′
ANP	Forward	5′-GGAGCCTGCGAAGGTCAA-3′
	Reverse	5′-TATCTTCGGTACCGGAAGCTGT-3′
BNP	Forward	5′-CAGAAGCTGGAGCTGATAAG-3′
	Reverse	5′-TGTAGGGCCTTGGTCCTTTG-3′
β-MHC	Forward	5′-GCTGTTATTGCAGCCATTG-3′
	Reverse	5′-TTCCTGTTGCCCCAAAATG-3′

### Measurement of cell surface area

F-actin staining was performed as previously described to measure cell surface area [47]. In brief, freshly prepared H9C2 cells were fixed with 4% paraformaldehyde for 15 min at room temperature. After the cells were rinsed with PBS three times, they were permeabilized with 0.5% Triton X-100 for 15 min at room temperature, washed three times with PBS, and incubated in the dark with 5 µg/mL phalloidin stain for 1 h at room temperature to enable the visualization of F-actin. After they were rinsed with PBS, the cells were photographed with a fluorescence microscope (IX71, Olympus, Tokyo, Japan). At least five randomly selected areas were chosen per dish, and 20 cells were randomly selected in each area. The areas of 2D cell surface of the selected H9C2 cells were quantified with ImageJ software (NIH, Bethesda, Md, USA).

Alpha-actinin staining was performed as previously described [48]. Briefly, the cells infected with LV-AKT1 were fixed with 4% paraformaldehyde and then permeabilized with 0.5% Triton X-100 for 15 min. Next, the cells were blocked with 5% bovine serum albumin (BSA) for 2 h at room temperature and then incubated with rabbit anti-sarcomeric alpha-actinin antibody overnight at 4 °C. The cells were then incubated with an Alexa Fluor 594 conjugated goat anti-rabbit IgG secondary antibody and observed by confocal microscopy.

### Western blot analysis

Western blotting was performed as described previously [49]. Briefly, cells were lysed with chilled RIPA buffer (Catalog number WB009, GUOAN, Xi’an, China) supplemented with protease inhibitor, and the insoluble components were removed by centrifugation at 12,000×*g* for 15 min at 4 °C. Then, the protein concentrations were measured with the BCA method. After denaturation, the total proteins were separated by 15% or 10% SDS-PAGE and transferred onto 0.2 µm or 0.45 µm polyvinylidene fluoride membranes. After they were blocked with 5% BSA for 2 h at room temperature, the membranes were incubated with primary antibodies diluted in blocking buffer overnight at 4 °C. The membranes were washed three times with TBST and further incubated with HRP-conjugated secondary antibodies for 2 h at room temperature. After the membranes were washed three times with TBST, an enhanced chemiluminescence (ECL) substrate was added, and the target protein bands were visualized with a ChemiDoc™ XRS+ system (Bio-Rad, Hercules, California, USA). ImageJ (version 1.46) was used to compare the gray values of the target bands, and GAPDH served as an internal control.

### Construction of AKT1 overexpressing stable cell line

Lentiviral vectors to overexpress AKT1 were constructed as previously described [50]. The complete ORF of rat *AKT1* was inserted into the pCDH-CMV-MCS-EF1-GreenPuro vector, and then, this construct was transfected into HEK293T cells together with the pCMV-VSV-G and pCMV-dR8.91 plasmids. The lentivirus particles were collected at 48 h post-transfection. A total of 1 × 10^5^ H9C2 cells in one well of a 24-well plate were infected with AKT1 lentivirus at 1.2 × 10^5^ TU and then cultured in high-glucose DMEM containing 10% FBS. After 48 h, the infected cells were subjected to puromycin selection at the concentration of 5 μg/mL. A single clone of H9C2 was obtained after approximately 10 days of puromycin selection. The expression of AKT1 was evaluated by qPCR and western blot.

### Measurement of intracellular calcium concentration ([Ca^2+^])

The measurement of the intracellular calcium concentration was performed as previously described [51]. In brief, the cells were washed three times with Tyrode’s salt solution. Then 500 µL of Fluo-3 acetoxymethyl ester (Fluo-3/AM, 5 μmol/L) was added to the cells, and the cells were cultured for 30 min at 37 °C in a 5% CO_2_ atmosphere in the dark. At the end of the incubation period, 1 mL of Tyrode’s salt solution was used to gently wash the cells three times. Finally, 1 mL of Tyrode’s salt solution was added to each group of cells and incubated for 20–30 min in the dark to ensure complete AM esterification in the cells. The fluorescence intensity was measured by laser scanning confocal microscopy using an excitation wavelength of 488 nm and an emission wavelength of 525 nm. The maximum fluorescence signal was recorded by fixed field photography, and the signal was input into the computer system through the photomultiplier tube and a digital afferent camera. The average intracellular fluorescence intensity was calculated by ImageJ software to determine the intracellular Ca^2+^ level.

### Statistical analysis

GraphPad Prism 7.0 (GraphPad Software Inc., La Jolla, CA) was used for statistical analysis. Statistical significance was determined by one-way ANOVA with Bonferroni correction for multiple comparisons or by unpaired Student’s t-test. A value of P < 0.05 was considered to indicate statistical significance. The number of independent experiments performed is indicated in the figure legends.

## Supplementary information

**Additional file 1. Fig. S1**: P-AKT1 (Thr308) was downregulated in the AVP-induced hypertrophic H9C2 cells. **Fig. S2**: AKT1 overexpression upregulated the protein expression of SERCA2 and downregulated the protein expression of PLN.

## Data Availability

The datasets used and/or analyzed during the current study are available from the corresponding author on reasonable request.

## Abbreviations

AVP: Arginine vasopressin
Met: Metoprolol
SR: Sarcoplasmic reticulum
SERCA2: Sarcoplasmic/endoplasmic reticulum Ca^2+^-ATPase 2
PLN: Phospholamban
HF: Heart failure
ANP: Atrial natriuretic peptide
BNP: B-type natriuretic peptide
β-MHC: Beta-myosin heavy chain
