# Supplementary material for: Metoprolol alleviates arginine vasopressin-induced cardiomyocyte hypertrophy by upregulating the AKT1–SERCA2 cascade in H9C2 cells
Source: Cell Biosci. 2020 May 24;10:72. doi: 10.1186/s13578-020-00434-y (PMC7247229; doi:10.1186/s13578-020-00434-y)
Supplement: Supplementary file 1 — Additional file 1. Fig. S1: P-AKT1 (Thr308) was downregulated in the AVP-induced hypertrophic H9C2 cells. Fig. S2: AKT1 overexpression upregulated the protein expression of SERCA2 and downregulated the protein expression of PLN. [file 13578_2020_434_MOESM1_ESM.docx]

**Additional file**

**Metoprolol** **alleviates Arginine vasopressin-induced** **cardiomyocyte hypertrophy by upregulating the AKT1-SERCA2 cascade in H9C2 cells**

Jieqiong Zhao, Yonghong Lei, Yanping Yang, Haibo Gao, Zhongchao Gai, Xue Li

**Inventory of supplemental information:**

**Fig. S1**: P-AKT1 (Thr308) was downregulated in the AVP-induced hypertrophic H9C2 cells.

**Fig. S2**: AKT1 overexpression upregulated the protein expression of SERCA2 and downregulated the protein expression of PLN.


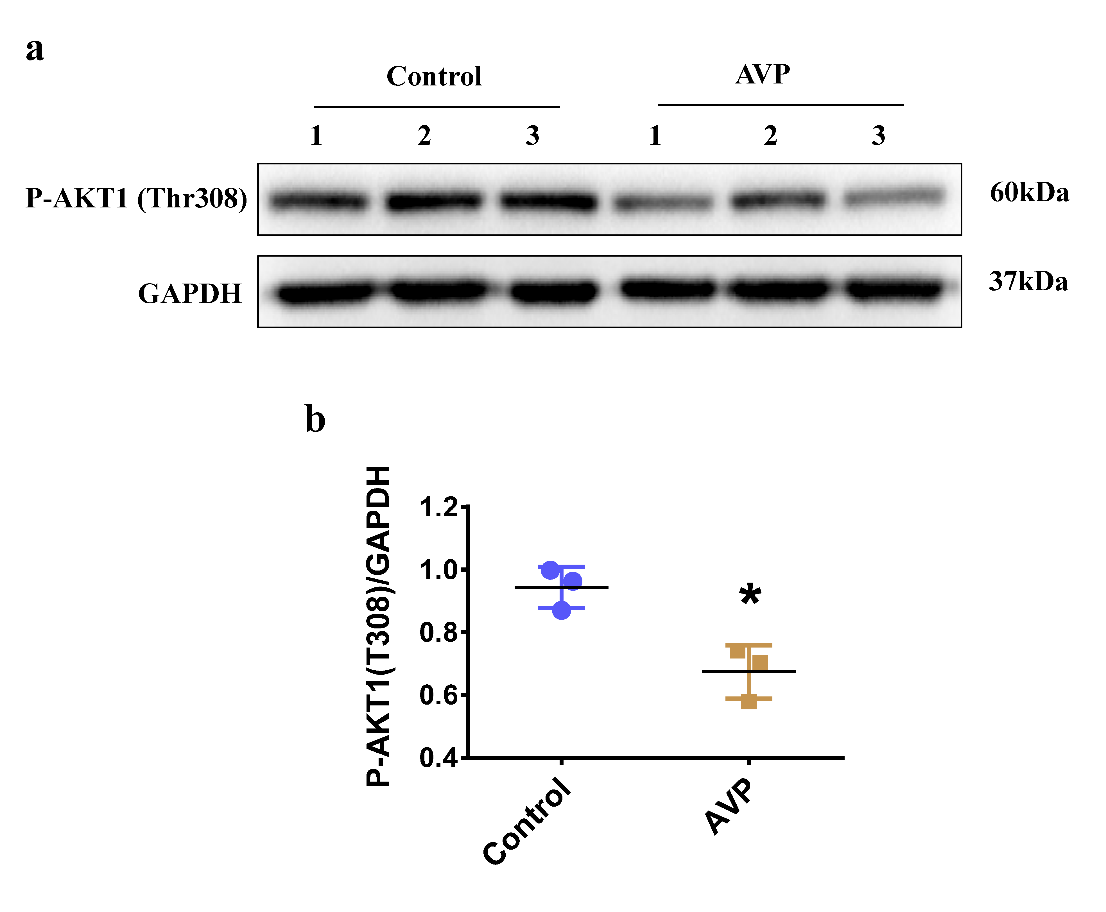


**Fig. S1:** **P-AKT1 (Thr308) was downregulated in the AVP-induced hypertrophic H9C2 cells.** (a) The phosphorylation of AKT1 at Thr308 was detected by western blot. GAPDH was taken as the loading control. (b) Quantification of the protein abundances in (a). Data are presented as the mean ± S.E.M. of three independent experiments (labeled as 1, 2 and 3). * indicates P< 0.05.


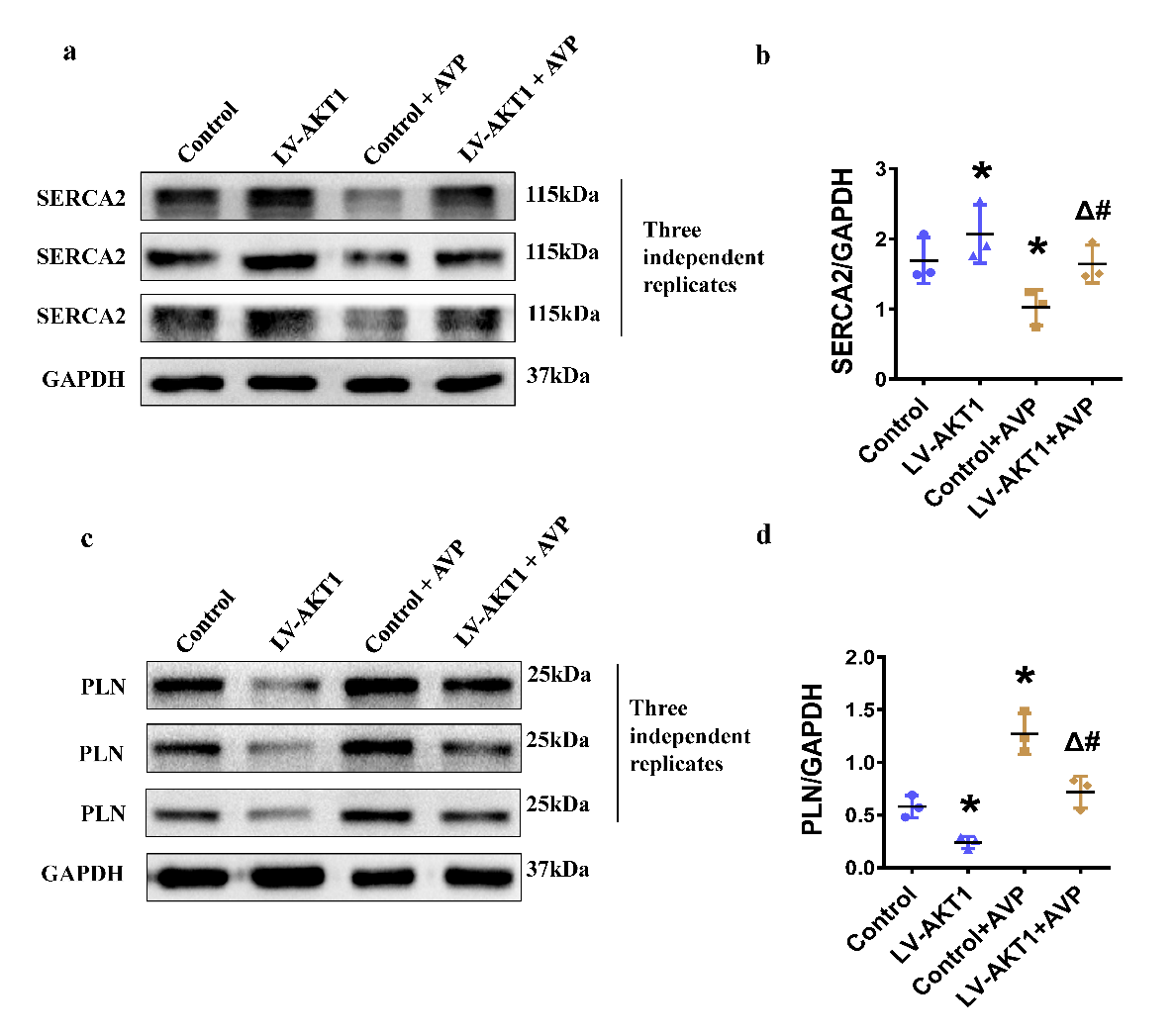


**Fig. S2:** **AKT1 overexpression upregulated the protein expression of SERCA2 and downregulated the protein expression of PLN**. (a, b) The protein expression and quantification of the SERCA2 in the AKT1 overexpressing stable strain. (c, d) The protein expression and quantification of the PLN in the AKT1 overexpressing stable strain. GAPDH was used as the loading control. All data are presented as the mean ± S.E.M. of three independent experiments. *, #, Δ both indicates P < 0.05. * compared with Control; # compared with Control + AVP; Δ compared with LV-AKT1.
